# Involvement of GLR-mediated nitric oxide effects on ROS metabolism in Arabidopsis plants under salt stress

**DOI:** 10.1007/s10265-024-01528-1

**Published:** 2024-03-06

**Authors:** Azime Gokce, Askim Hediye Sekmen Cetinel, Ismail Turkan

**Affiliations:** Department of Biology, Faculty of Science, Ege University, Bornova, Izmir, 35100 Turkey

**Keywords:** Antioxidant enzymes, Glutamate receptors, Nitric oxide, Reactive oxygen species, Salt stress

## Abstract

Plant glutamate receptor-like channels (GLRs) play important roles in plant development, immune response, defense signaling and Nitric oxide (NO) production. However, their involvement in abiotic stress responses, particularly in regulating Reactive Oxygen Species (ROS), is not well understood. This study aimed to investigate GLR-mediated NO production on ROS regulation in salt-stressed cells. To achieve this, *Arabidopsis thaliana* Columbia (Col-0) were treated with NaCl, glutamate antagonists [(DNQX (6,7-dinitroquinoxaline-2,3-dione and AP-5(D-2-amino-5-phosphono pentanoic acid)], and NO scavenger [cPTIO (2-(4-Carboxyphenyl)-4,4,5,5-tetramethylimidazoline-1-oxyl-3-oxide potassium salt)]. Salt-stressed plants in combination with DNQX and AP-5 have exhibited higher increase in lipid peroxidation (TBARS), hydrogen peroxide (H_2_O_2_) and superoxide radical (O^−2^) contents as compared to solely NaCl-treated plants. Furthermore, NO and total glutathione contents, and S-nitrosoglutathione reductase (GSNOR) activity decreased with these treatments. AP-5 and DNQX increased the activities of NADPH oxidase (NOX), catalase (CAT), peroxidase (POX), cell wall peroxidase (CWPOX) in salt-stressed Arabidopsis leaves. However, their activities (except NOX) were significantly inhibited by cPTIO. Conversely, the combination of NaCl and GLR antagonists, NO scavenger decreased the activities of ascorbate peroxidase (APX), superoxide dismutase (SOD), glutathione reductase (GR), dehydroascorbate reductase (DHAR) and monodehydroascorbate reductase (MDHAR) resulting in elevated GSSG levels, a low GSH/GSSG ratio, impaired ROS scavenging, excessive ROS accumulation and cell membrane damage. The findings of this study provide evidence that GLR-mediated NO plays a crucial role in improvement of the tolerance of Arabidopsis plants to salt-induced oxidative stress. It helps to maintain cellular redox homeostasis by reducing ROS accumulation and increasing the activity of SOD, GSNOR, and the ASC-GSH cycle enzymes.

## Introduction

In the animal system, glutamate (Glu), an important excitatory neurotransmitter, has been reported to be involved in growth processes in plants (Forde 2014; Reiner and Levitz 2018). Glu, also later known as G-protein coupled receptors, exerts its signaling role in presynaptic cells generally by two types of receptors: ionotropic glutamate receptors (iGluRs) and metabolic glutamate receptors (mGluRs) (López-Bucio et al. 2019; Reiner and Levitz 2018). In animals, mGLuRs are glutamate receptors belonging to the superfamily of G-protein coupled proteins, while iGluRs are N-methyl-D-aspartate (NMDA), kainate and α-amino-3-hydroxy-5 methylisoxazole-4-propionic acid (AMPA) including Na^+^, K^+^ and Ca^2+^ consists of non-selective cation channels (Dingledine et al. 1999; Naz et al. 2022). Ionotropic glutamate receptors (iGluRs) have been identified in all three domains of life, and homologues of iGluRs known as Glutamate receptor-like (GLR) channels have also been found in the genomes of Chlamydomonas, chlorophytum, ferns, moss, gymnosperms, and flowering plants (De Bortoli et al. 2016). Plants show high similarity in nucleotide and amino acid sequences to mammalian iGluRs (Lacombe 2001) and thus have a family of so-called glutamate receptor-like channels (GLRs) (Lam et al. 1998; Price et al. 2012).

Recently, plant GLRs have been shown to play a role in cellular processes such as; abscisic acid (ABA) biosynthesis and signaling (Kang and Turano 2003; Kang et al. 2004; Kong et al. 2015), carbon and nitrogen metabolism (Kang and Turano 2003; Kang et al. 2004), water loss (Kang et al. 2004; Lu et al. 2014), in lateral root formation (Vincill et al. 2012), root gravitropism (Miller et al. 2010a, b), root development (Singh et al. 2016), innate immune responses (Forde and Roberts 2014; Kang et al. 2004; Kwaaitaal et al. 2011; Li et al. 2013; Manzoor et al. 2013), stomatal closure (Cho et al. 2009), seed germination (Kong et al. 2015), pollen tube growth (Michard et al. 2011; Wudick et al. 2018), leaf-to-leaf electrical signal transmission from wound (Mousavi et al. 2013) and plant defense response against aphids (Vincent et al. 2017). Subsequently, sequence similarities between model plant *Arabidopsis thaliana* (L.) Heynh. Col-0 GLRs and animal iGluRs were noted (Lam et al. 1998). 20 genes encoding subunits of glutamate-like receptors (AtGLRs) were identified in the genome of this plant [divided into 3 protein families (Clade I, Clade II, and Clade III) (Chiu et al. 2002; Davenport 2002)] (Lacombe et al. 2001; Lam et al. 1998). The role of GLRs in adapting to environmental stresses has also been reported. For example, AtGLRs mediate [Ca^2+^]cyt fluctuation, regulating the stress adaptation process and defense responses, apart from many developmental processes (Manzoor et al. 2013; Singh et al. 2016). In a recent study, it was shown that atglr3.4 and atglr3.7 T-DNA insertion mutants are hypersensitive to salt stress, possibly by regulating Ca^2+^ flux during seed germination (Cheng et al. 2016).

Salt stress is one of the main global agricultural problems affecting crop productivity by causing ionic and osmotic stress (van Zelm et al. 2020). Overproduction of ‘Reactive Oxygen Species’ (ROS) takes place under salt stress, which can have a detrimental effect on DNA, RNA, proteins and lipids, thereby causing oxidative stress (Foyer and Noctor 2005; Jaspers and Kangasjärvi 2010). ROS molecules such as hydroxyl radical (OH^.^), superoxide radical (O^−2^) and hydrogen peroxide (H_2_O_2_) are produced in lower concentrations during normal metabolism and developmental progress. They play an important role as signal molecules in processes such as germination, development, differentiation and redox regulation (Mittler 2002). Stress occurs when the delicate balance between ROS production and scavenging is disrupted. For this reason, plants use different defense mechanisms to cope with salt stress. The antioxidant defense system is at the forefront of these. The accumulation of ROS resulting from stress is detoxified by enzymatic [superoxide dismutase (SOD), catalase (CAT), ascorbate peroxidase (APX), glutathione reductase (GR), glutathione peroxidases (GPX) and glutathione S-transferase (GST)] and non-enzymatic [ascorbate (ASC), glutathione (GSH), α-tocopherol, carotenoids and flavonoids] antioxidants (Gill et al. 2011; Mitler et al. 2004). In recent times, in the literature, the term ‘Reactive Nitrogen Species’ (RNS) has been used to donate nitric oxide (NO) and other NO-related molecules such as S-nitrosoglutathione (GSNO), S-nitrosothiols (RSNOs) and peroxynitrite (ONOO–), which are formed as a result of the interaction of ROS and NO (Corpas et al. 2007). Plants have a similar set of plant defenses against RNSs as ROSs. Apart from antioxidant enzymes involved in NO scavenging, another important enzyme is S-nitrosoglutathione reductase (GSNOR) (Leterrier et al. 2011). NO, an important RNS molecule, is another signaling molecule that has emerged in recent years, and has been shown to play a wide variety of roles in a/biotic stresses as well as in different physiological and developmental processes (Kolbert et al. 2019; Neill et al. 2008). NO operates and produces in various parts of the cell by several oxidative and reducing pathways (Gupta et al. 2021). One study identified NO as a key component in ABA-induced stomatal closure under drought stress (García-Mata and Lamattina 2003). NO has also appeared as a key player in plant resistance responses to pathogens (Mur et al. 2006). Interestingly, one of the recent studies showed that putative plant GLRs participate in calcium flux and NO production induced by the fungal elicitor cryptogein (Vatsa et al. 2011). In another study, the effect of GLR-related NO exchange was investigated using glutamate antagonists (DNQX- 6,7-dinitroquinoxaline-2,3-dione and AP-5-D-2-amino-5-phosphonopentanoic acid) in adaptation to short-term water stress during seedling formation in *Medicago truncatula*. The plant GLR, as has been shown to be, involved in defence signaling with mechanisms homologous to mammals and may also play a role in NO production, suggesting that these channels are required in defence responses as they are involved in NO production (Philippe et al. 2019). However, there is no information on the effects of GLR-mediated NO production on ROS regulation of plants under abiotic stress. Thus the aim of our study was to evaluate the effects of foliar application of glutamate compatative antagonists (DNQX and AP-5) and NO scavenger (cPTIO) on the contents of H_2_O_2_, O^2−^, and NO, lipid peroxidation level, the activities of NADPH oxidase, some antioxidant enzymes (SOD, CAT, POX, CWPOX) and ASC-GSH cycle enzyme activites (APX, GR, MDHAR, DHAR), GSNOR activities and glutathione pool (GHS/GSSG) of *A. thaliana* wild type Columbia (Col) ecotype under salt stress.

## Materials and methods

### Plant material, growth conditions, and stress application

The Arabidopsis ecotype Columbia (Col-0) was obtained from the Arabidopsis Information Resource (TAIR, www.arabidopsis.org). Seeds were sterilized using 70% ethanol (1 min) and 4% sodium hypochlorite (NaOCl) solution (10 min). Plants were grown in a plant growth chamber (JSR, JSPC-420C, Korea) by usage of a hydroponic system under controlled conditions (16/8 h light/dark cycle, 23/21 °C, relative humidity 60% and light intensity 200 μmol photon m^−2^ s^−1^) with half-strength Hoagland’s solution. After a 3-week growth period, the plants were exposed to 100 mM NaCl for salt treatment and were was added to Hoagland’s solution. Apart from that, glutamate receptor antagonists with 0.1 mM AP-5 (D-2-amino-5-phosphono pentanoic acid; Sigma-Aldrich) and 0.5 mM DNQX (6,7-dinitroquinoxaline-2,3-dione) were applied to plant leaves by spraying. Afterward, the plants were harvested at 48 h and 96 h of the treatment. In many studies on glutamate receptors, antagonist applications are made in different concentrations, for different periods of time and different plants (Cheng et al. 2018; Li et. al. 2019; Manzoor et al. 2013; Michard et al. 2011; Philippe et al. 2019; Sivaguru et al. 2003; Teardo et al. 2006; Vatsa et al. 2011; Yoshida et al. 2016). According to these studies, various concentrations of DNQX and AP-5 (1 mM, 250 μM, 400 μM, 500 μM DNQX and 50 μM, 100 μM, 200 μM, 1 mM, 2 mM etc.) and time courses (2 h, 24 h, 48 h etc.) were used in our preliminary studies. We observed a significant difference at 0.1 mM AP-5 and 0.5 mM DNQX and 48 h and 96 h as application times. The most commonly used agents as Glutamate Receptor antagonists are DNQX and AP-5. DNQX was used as competitive AMPA glutamate receptor antagonist. AP-5 was used as selective and competitive NMDA antagonist (Coccurello et al. 2012). Furthermore, the inhibitory effect of DNQX on all AtGLRs is assumed to rely on binding within the ligand binding sites, whereas AP-5 is known to compete with the natural ligand, possibly at the L-glutamate binding site (Dubos et al. 2003). DNQX and AP-5 have similar effects within the cell. For example, cytosolic Ca^2+^ is suppressed by both DNQX and AP-5. However, the highest suppression occurred with AP-5 (Vatsa et al. 2011). Moreover, the duration of salt and antagonists treatment was chosen by considering the percentages of green seedlings of genotypes that survived. Additionally, the harvested plants were frozen in liquid nitrogen and stored at –80 °C until further enzymatic analysis.

### Shoot fresh weight

Shoot fresh weights of six plants were measured randomly from each group of harvested plants.

### Relative water content (RWC)

First, five leaves from each species were harvested and their FW was determined. The leaves were floated on deionized water for 6 h under low irradiance. The turgid leaves were rapidly blotted to remove excess water and their turgid weights (TWs) were measured. The DW was measured right after the leaves were dried within an oven. The relative water content (RWC) was calculated:

$${\text{RWC }}\left( \% \right) \, = \, \left[ {\left( {{\text{FW }} - {\text{ DW}}} \right)/\left( {{\text{TW }} - {\text{ DW}}} \right)} \right] \, \times { 1}00$$

### Determination of NO content

The NO presence, to be determined according to Zhou et al. 2005, was homogenized with 3 ml of 0.6 g leaf sample, 50 mM cold acetic acid containing 4% zinc diacetate and pH: 3.6. The obtained homogenates were centrifuged at 10,000 *g* at + 4 0C for 15 min, and after the supernatant was taken, the pellet was washed again with 1 ml of extraction buffer and centrifuged. 0.1 g of charcoal was added to the collected supernatants. The supernatants were then vortexed and filtered. 1 ml of Griess reagent was added to 1 ml of filtered sample and mixed, then incubated for 30 min at room temperature and the samples were measured at 540 nm wavelength.

### NADPH oxidase (NOX) activity

NOX (EC 1.6.3.1) activity was determined according to Jiang and Zhang (2002). The reaction mix contained 50 mM Tris–HCl buffer (pH 7.5), 0.5 mM sodium 3,3′-(− [(phenylamino)carbonyl]-3,4-tetrazolium)-bis(4-methoxy-6-nitro) benzene-sulfonic acid hydrate (XTT), 100 µM NADPH•Na4, and 20 µg of protein. After the addition of NADPH, the following XTT reduction was observed at 470 nm. The corrections of background production were determined in the presence of 50 U SOD. Activity was calculated using the extinction coefficient 2.16 × 104 M^−1^ cm^−1^. One unit of NOX activity was defined as 1 nmol ml^−1^ XTT oxidized min^−1^.

### *Determination of H*_*2*_*O*_*2*_* content*

The H_2_O_2_ assay was determined using the eFOX reagent (Cheeseman et al. 2006). The modified ferrous ammonium sulfate/xylenol orange (FOX) assay was preferred because of its sensitivity, stability, and adaptability to a large number of samples. Homogenization was performed using ice-cold acetone containing 25 mM H_2_SO_4_. Then samples were centrifuged for 5 min at 3000 × *g* at 4 °C. For 50 μl of the supernatant, 950 μl (250 μM ferrous ammonium sulfate, 100 μM sorbitol, 100 μM xylenol orange, 1% ethanol, v/v) of eFOX reagent was used. The reaction mixtures were incubated at room temperature for 30 min and absorbance at 550 and 800 nm was determined. H_2_O_2_ concentrations were calculated via a standard curve prepared with known concentrations of H_2_O_2_.

### *Determination of O*^*2.*−^* content*

The amount of superoxide anion radical was determined (Oracz et al. 2007). After 0.2 g of plant sample was homogenized in 4 ml of 50 mM Na-P buffer (pH 7.8) at 4 ºC, the homogenates were centrifuged at 16,000 *g* for 15 min. 1 ml of the supernatant was incubated in reaction buffer, 50 mM Na-P buffer containing 1 mM hydroxylamine, for 30 min at 25 ºC. Then, 0.5 ml 17 mM sulfanilamide and 0.5 ml 7 mM 2-naphtylamine were added over 0.5 ml of this reaction mixture and incubated for 30 min at 25 ºC. After incubation, the samples were centrifuged at 13,000 *g* for 10 min and measured at 540 nm.

### Enzyme extractions and assays

All assays were performed at 4 °C. For the extractions of protein and enzyme, 0.1 g of the sample was homogenized in 500 µl of 50 mM Tris–HCl (pH 7.8) containing 0.1 mM EDTA, 0.1% (w/v) Triton-X100, 1 mM phenylmethanesulfonyl fluoride (PMSF), and polyvinylpyrrolidone (PVP; 1%, w/v). Apart from this, the homogenization buffer that determinates APX activity was prepared with the addition of 5 mM ascorbate. Samples were centrifuged at 14,000 × *g* for 10 min, and supernatants were used to determinate protein content and enzyme activities. Total soluble protein content of the enzyme extracts was determined using BSA as a standard (Bradford 1976).

### Total SOD activity

The extracts will be separated electrophoretically with a 12.5% separation gel and a 5% alignment gel with a constant current (100 mA) at + 4 °C. SOD activity will be determined by riboflavin and nitrobluetetrazolium (NBT) dye according to Beauchamp and Fridovich (1973).

### Catalase (CAT) activity

CAT (EC 1.11.1.6) activity was determined by measuring the initial loss rate of H_2_O_2_ at 240 nm (Bergmeyer 1970). The reaction mixture contained 50 mM Na phosphate buffer (pH 7.0) with 0.1 mM EDTA and 3% H_2_O_2_. A reduction in absorption was then observed. One unit of CAT activity was defined as 1 mmol H_2_O_2_ oxidized ml^−1^ min^−1^.

### Peroxidase (POX) and cell wall peroxidase (CWPOX) activity

The activity of enzymes was measured according to Herzog and Fahimi (1973). The same homogenates were used to determine both activities, but different pretreatments were performed earlier. After the sample was homogenized, centrifugation was performed at 14,000 × *g* for 10 min at 4 °C and obtained homogenates were used for POX assay. And for the CWPOX assay, pellets were rewashed in 50 mM sodium phosphate (pH 5.8) and centrifuged at 1000 × *g* for 10 min at + 4 °C. Afterwards, pellets were resuspended in 1 ml dH_2_O and 1 M NaCl was pipetted into the tubes and the mixture stirred for 2 h. After that, the samples were centrifuged at 1000 rpm for 10 min. The reaction mixture containing 0.1% (w/v) gelatin, 150 mM Na phosphate citrate buffer (pH 4.4) and 3,3'-diaminobenzidine-tetra hydrochloride dihydrate solution that includes 0.6% H_2_O_2_ was used, and absorbance was measured at 465 nm. One unit of POX and CWPOX activities were defined as mmol H_2_O_2_ decomposed ml^−1^ min^−1^.

### Ascorbate peroxidase (APOX) activity

APX (EC 1.11.1.11) activity was measured by the method determined by Nakano and Asada (1981). The reaction mixture containing 50 mM K phosphate buffer (pH 7.0), 0.1 mM EDTA Na2, 0.5 mM ascorbate, 0.1 mM H_2_O_2_ was used, and absorbance was measured at 290 nm as ascorbate is oxidized. The concentration of the oxidized ascorbate was calculated by using the extinction coefficient of 2.8 mM^−1^ cm^−1^.

### Monodehydroascorbate reductase (MDHAR) activity

MDHAR activity was assessed as described by Arrigoni et al. (1981). MDHAR was measured with NADH oxidation in the presence of ascorbate oxidase (1 U) at 340 nm.

### Dehydroascorbate reductase (DHAR) activity

DHAR activity was determined via Nakano and Asada (1981). The reaction mixture contained 2.5 mM GSH, 0.2 mM DHA and 0.1 mM EDTA in a 50 mM K phosphate (pH 7.0). The measured absorbance value, 265 nm for 1 min, was elevated. One unit of DHAR activity was defined as 1 nmol DHA recycled ml^−1^ min^−1^.

### Glutathione reductases (GR) activity

GR (EC 1.6.4.2) activity was measured using a reaction mixture (Foyer and Halliwell 1976) that contains 25 mM Na phosphate buffer (pH 7.8), 0.5 mM GSSG, 0.12 mM NADPH, Na4 and 0.1 ml enzyme extract in a final assay volume of 1 ml is according to NADPH oxidation followed at 340 nm. Activity was calculated using the extinction coefficient of NADPH (6.2 mM^−1^ cm^−1^). One unit of GR was defined as 1 mmol ml^−1^ GSSG reduced min^−1^.

### Lipid peroxidation

Lipid peroxidation [reflected by the content of thiobarbituric acid reagent (TBARS)] was measured by the leaf tissue homogenized with (200 mg) in 800 µl of cold 5% (w/v) trichloroacetic acid. Centrifugation was made at 12,000 × *g* for 30 min and further processed according to the method described by Madhava Rao and Sresty (2000). The TBARS concentration was calculated using an extinction coefficient of 155 mM cm^−1^.

### Reduced glutathione (GSH) and oxidised glutathione (GSSG) content

Contents of the non-enzymatic antioxidants were determined according to Queval and Noctor (2007). Extraction was performed at 4 ^◦^C. Leaf tissue (0.1 g) was homogenized with 1 ml 0.2 N HCl and centrifugation was performed at 16 000 × *g* for 10 min. The supernatant (0.5 ml) was neutralized with approximately 0.4 ml 0.2 M NaOH in the presence of 50 μl 0.2 M NaH_2_PO_4_ (pH 5.6). Whereas the glutathione content was measured at 340 nm, GSSG content was measured using 2-vinylpyridine derivatization after an enzyme cycling assay at 340 nm.

### S-nitrosoglutathione reductase (GSNOR) enzyme activity

Harvested plant leaves were homogenized with homogenization buffer containing 20 mM Tris–HCl (pH:7.5), 0.2 mM NADH, 2 mM dithiothreitol (DTT), 0.5 mM EDTA, 0,2% Triton X-100 and 10% glycerol. After the homogenates are centrifuged at 27,000 *g* for 25 min at 4 °C, the reaction begins with the addition of 0.4 mM GSNO. GSNOR activity was observed spectrophotometrically by monitoring NADH oxidation at 25 °C at 340 nm. Activity was expressed as NADH consumed per minute in nmol per 1 mg of protein (Barroso et al. 2006; Sakamoto et al. 2002).

### Statistical analysis

The experiments were repeated two times and biological triplicates were used from each experiment for all analyses (*n* = 6). The results were expressed as the mean ± standard error of the mean. The normality of the groups was tested with the Shapiro–Wilk test, followed by a one-way ANOVA. Whether the variance between groups was equal-homogeneous was determined by Levene's test. According to the result of the equality of variance between the groups, the differences in-between groups were determined by Tukey test if the variances were equal, and if not the Games-Howell test was used.

## Results

### Shoot fresh weight

Shoot fresh weight, used as a parameter for plant growth promoting actions, were measured in GLR antagonist-treated and –untreated groups in order to understand the extent of the GLR improved tolerance against salt stress and how it affected growth (Table 1). At 48 h, AP-5 and DNQX did not affect the shoot fresh weight compared with the control (Table 1). Shoot fresh weight elevated approximately fourfold until 96 h under normal conditions. However, DNQX, NaCl, NaCl + DNQX and NaCl + cPTIO-treated Arabidopsis plants showed 44%, 44%, 60% and 54% reduction in shoot fresh weight respectively. Moreover, the greatest decrease in shoot fresh weight was observed in solely AP-5 (73%) and NaCl + AP-5 (82%) treated-plants at 96 h (Table 1).

**Table 1:** Effect of salt stress (100 mM NaCl), GLR antagonists (0.5 mM DNQX and 0.1 mM AP-5) and NO scavenger (0.5 mM cPTIO) application on shoot fresh weight (g per plant), and relative water content (%) of Arabidopsis Col-0 ecotype

	Shoot fresh weight (g)	Relative water content (%)
48 h control	0.68 ± 0.026a	85.33 ± 2.16a
48 h DNQX	0.93 ± 0.01a	71.66 ± 0.13b
48 h AP-5	0.59 ± 0.03a	84.45 ± 0.13a
96 h control	1.55 ± 0.028b	87.88 ± 3.43a
96 h DNQX	0.86 ± 0.05a	77.18 ± 5.29c
96 h AP-5	0.41 ± 0.006c	86.21 ± 1.02a
96 h NaCl	0.86 ± 0.014a	70.53 ± 1.72b
96 h NaCl + DNQX	0.55 ± 0.02a	76.05 ± 11.01c
96 h NaCl ± AP-5	0.26 ± 0.11d	72.73 ± 3.60b
NaCl ± cTPIO	0.71 ± 0.004a	62.88 ± 1.55d

Data were analyzed with Jamovi 2.3.21 using Tukey’s multiple-range test at a significance level of *p* < 0.05, and different letters (a–d) indicate a significant difference between treatments of Data were expressed as the mean ± standard error of three independent biological replicates

### RWC

At 48 h, as compared to the control, the lowest amount of RWC was seen in the DNQX-treated plants with a decrease of approximately 16%, while no significant change was observed with AP-5 application. Similarly, at 96 h, AP-5 did not affect the RWC as compared to control. However, a decrease of 12% with DNQX, 19% with 96 h NaCl, 18% with NaCl + AP-5, and finally 28% with NaCl + cPTIO were determined, compared with control groups (Table 1).

### NO content

**Fig. 1:**
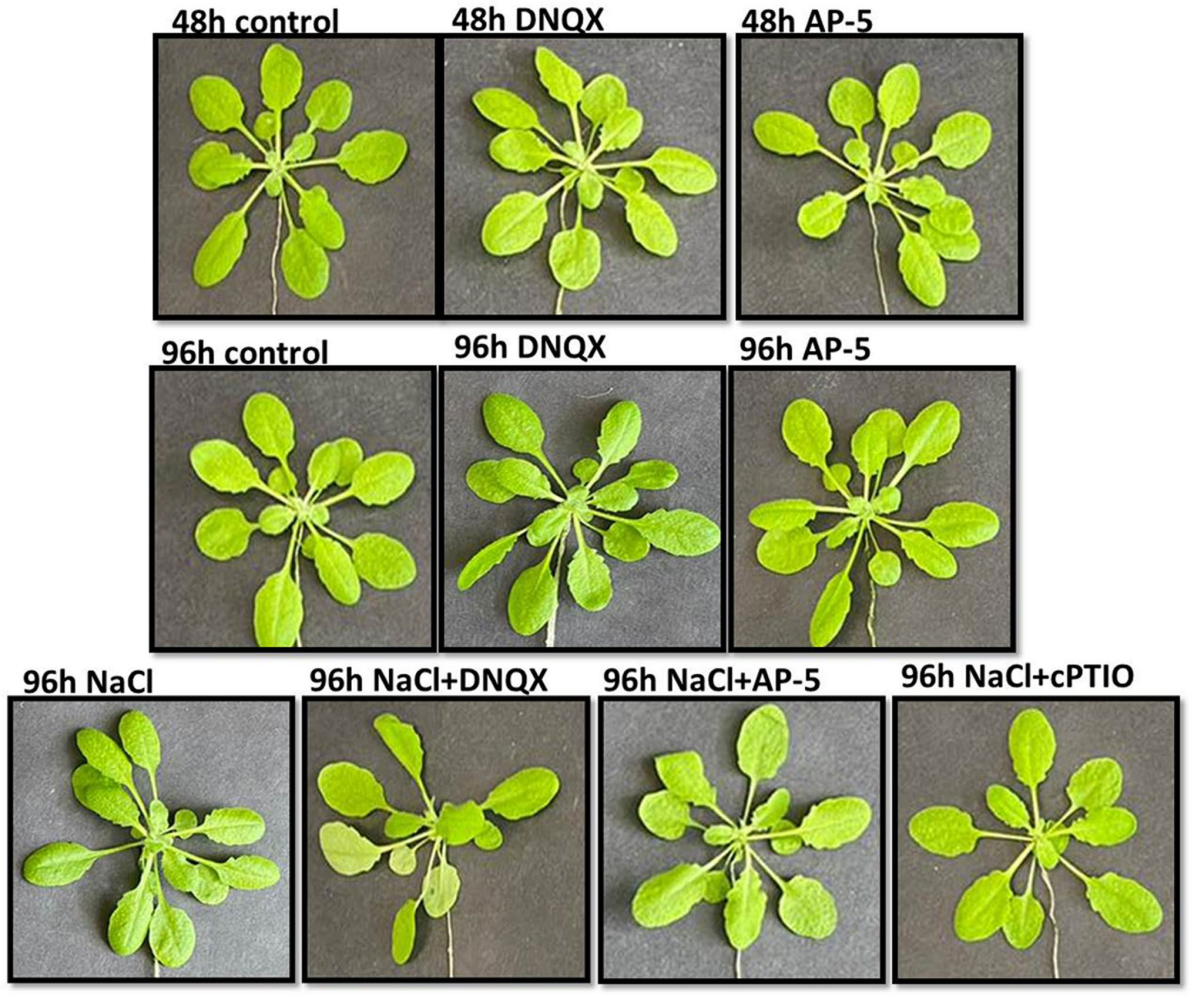
Four-week-old Arabidopsis Col-0 ecotype exposed to 100 mM NaCl, GLR antagonists (0.5 mM DNQX and 0.1 mM AP-5) and NO scavenger (0.5 mM cPTIO) for 48 h and 96 h

**Fig. 2:**
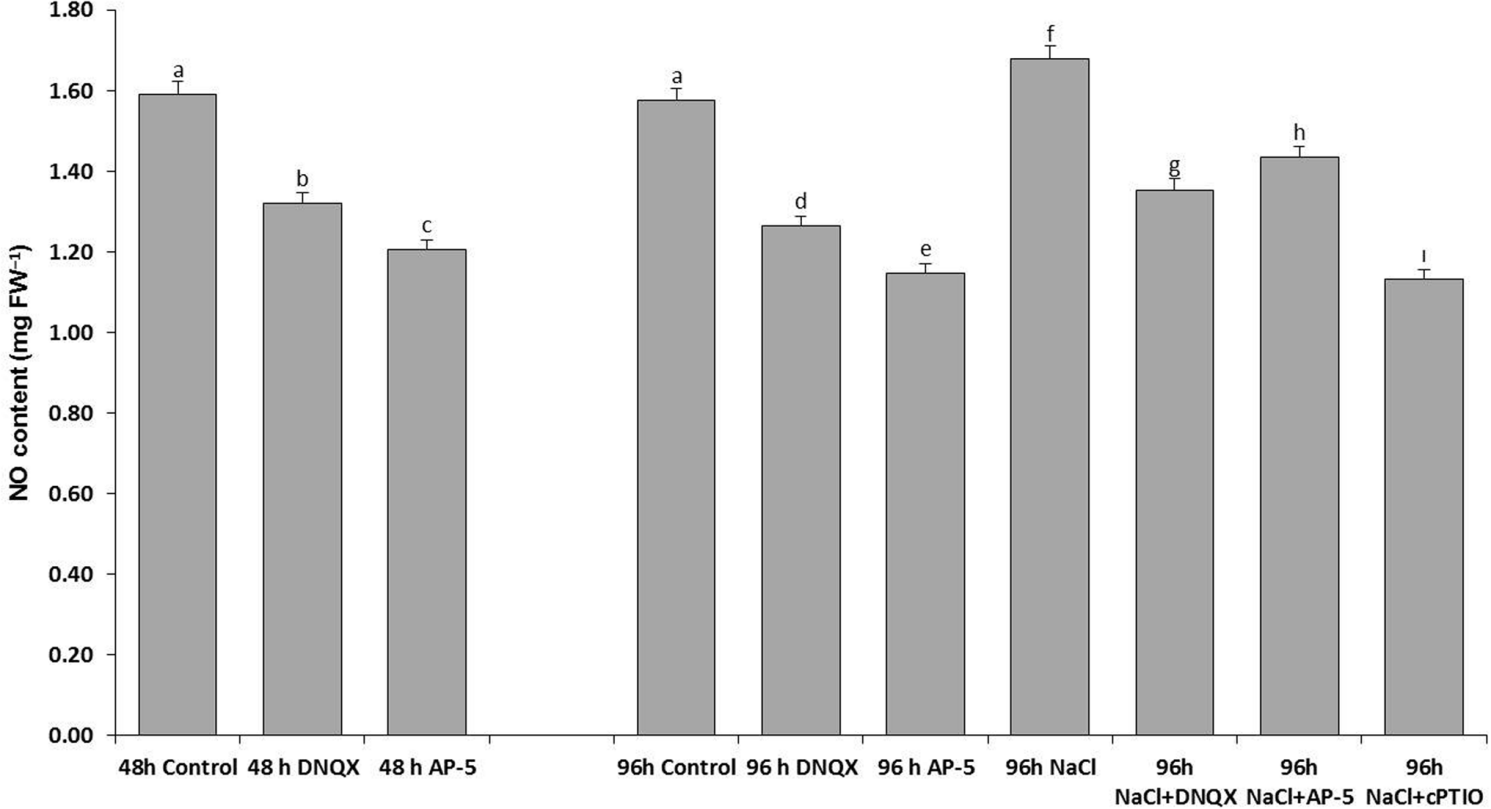
Effect of salt stress (100 mM NaCl), GLR antagonists (0.5 mM DNQX and 0.1 mM AP-5) and NO scavenger (0.5 mM cPTIO) application on NO content of Arabidopsis Col-0 ecotype. Data were analyzed with Jamovi 2.3.21 using Tukey’s multiple-range test at a significance level of *p* < 0.05, and different letters (**a**–**d**) indicate a significant difference between treatments of Data were expressed as the mean ± standard error of three independent biological replicates

To determine whether GLR antagonists AP-5 and DNQX affect the NO levels in the leaf cells of *A. thaliana Col-0* under normal and salt stress conditions, the NO levels in the leaves of plants subjected to pre-treatment with these antagonists under stress were measured. Subsequently, the NO levels in plants treated with DNQX and AP-5 under salt stress were compared with those of plants exposed only to salt stress and under normal conditions. At 48 h, GLR compatative antagonists, DNQX and AP-5, caused a significant decrease in NO content in solely DNQX and solely AP-5 treated-plants as compared to that of control. Similarly, at 96 h, NO content was decreased by alone DNQX (20%) and solely AP-5 treatments (28%) as compared to control. However, alone NaCl treatment increased NO content by %7 as compared to control (Fig. 1). At 96 h, the highest NO content was observed in solely NaCl-treated plants. On the other hand, NO content was decreased by 20%, 14% and 30% in the plants which were subjected to the combination of salt stress and DNQX, AP-5 and cPTIO, respectively, as compared to solely NaCl-treated plants. The lowest NO content was observed in NaCl + cPTIO treated-plants (Fig. 2).

### NOX activity

**Fig. 3:**
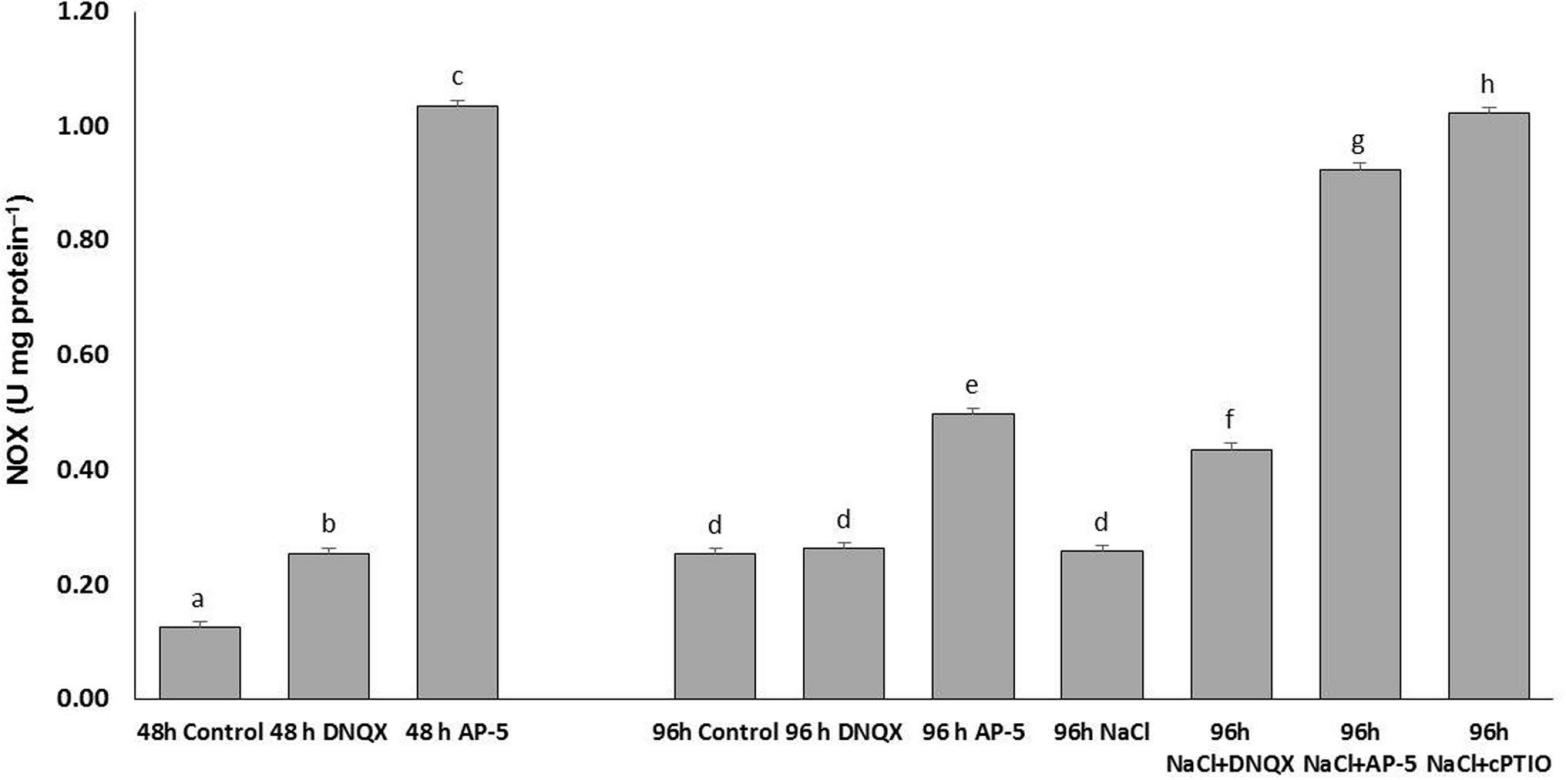
Effect of salt stress (100 mM NaCl), GLR antagonists (0.5 mM DNQX and 0.1 mM AP-5) and NO scavenger (0.5 mM cPTIO) application on NOX activity of Arabidopsis Col-0 ecotype. Data were analyzed with Jamovi 2.3.21 using Tukey’s multiple-range test at a significance level of *p* < 0.05, and different letters (**a**–**d**) indicate a significant difference between treatments of Data were expressed as the mean ± standard error of three independent biological replicates

Previous studies have revealed that salt stress leads to ROS production through NOX activation in the plant plasma membrane. For this reason, in this study, we determined the change in GLR-mediated NO production NOX activity and ROS contents such as H_2_O_2_ and O_2_^−^. A very large increase in NOX activity was seen with antagonist application at 48 h. While this amount of increase was twofold in the DNQX group, it increased approximately eightfold with the AP-5 application. At 96 h, NOX activity remained at the control level in the solely DNQX-treated plants. However, AP-5 caused a significant rise (twofold) in the NOX activity. On the other hand, the level of NOX activity in the NaCl-treated plants was at the same level as the control group, but DNQX, AP-5 and cPTIO increased the NOX activity by 1.69, 3.5 and 3.9 fold respectively in plants under salt stress as compared to solely NaCl-treated plants (Fig. 3).

### *H*_*2*_*O*_*2*_* content*

**Fig. 4:**
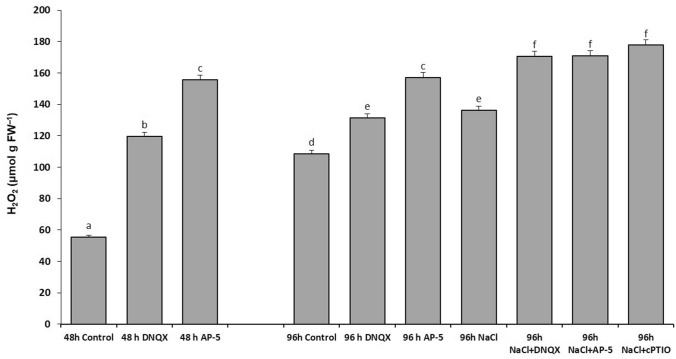
Effect of salt stress (100 mM NaCl), GLR antagonists (0.5 mM DNQX and 0.1 mM AP-5) and NO scavenger (0.5 mM cPTIO) application on H_2_O_2_ of Arabidopsis Col-0 ecotype. Data were analyzed with Jamovi 2.3.21 using Tukey’s multiple-range test at a significance level of *p* < 0.05, and different letters (**a**–**d**) indicate a significant difference between treatments of Data were expressed as the mean ± standard error of three independent biological replicates

DNQX and AP-5 treatments caused a significant increase in H_2_O_2_ content at 48 h and 96 h, as compared to control. Furthermore, at 96 h, salt stress increased H_2_O_2_ content by 44% in AP-5-treated plants, as compared to solely salt stressed plants. Similarly, in 100 mM NaCl DNQX-treated plants, salt stress increased the H_2_O_2_ content by 23% as compared to NaCl treated plants alone. Moreover, in the presence of salt, the H_2_O_2_ content was similarly increased by approximately 60% with the application of DNQX as compared to the control (Fig. 4).

### *O*^*2−*^* content*

At 48 h, the O^2−^ content increased twofold and 2, fivefold in DNQX and AP-5 treatments, respectively, compared with the control group. Similarly, at 96 h, both GLR antagonist treatments caused a significant increase in DNQX (44%) and AP-5 (38%) treated plants alone as compared to that of control.

**Fig. 5:**
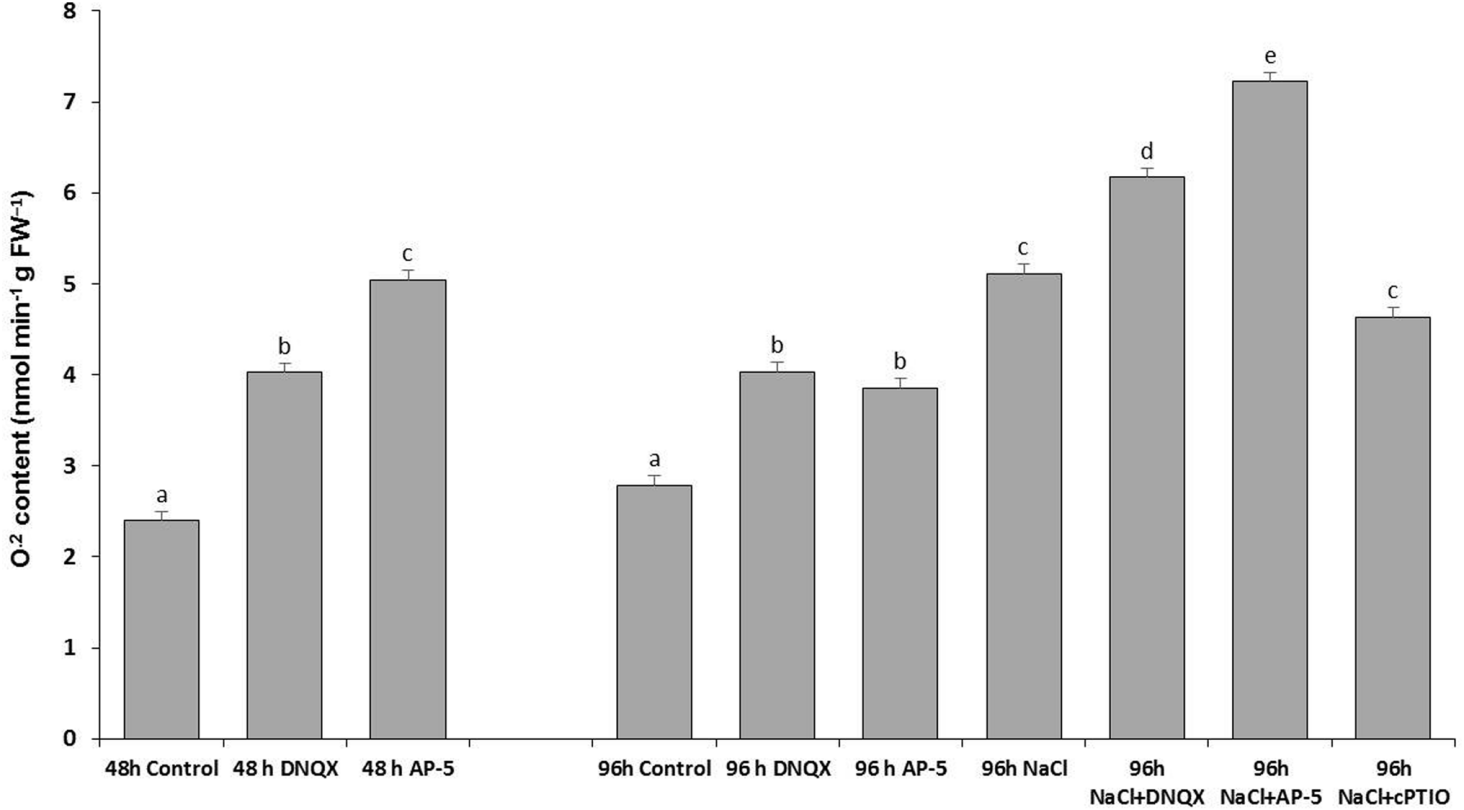
Effect of salt stress (100 mM NaCl), GLR antagonists (0.5 mM DNQX and 0.1 mM AP-5) and NO scavenger (0.5 mM cPTIO) application on O^.2^ of Arabidopsis Col-0 ecotype. Data were analyzed with Jamovi 2.3.21 using Tukey’s multiple-range test at a significance level of *p* < 0.05, and different letters (**a**–**d**) indicate a significant difference between treatments of Data were expressed as the mean ± standard error of three independent biological replicates

NaCl application increased the O^2−^ content by 83% compared to the control group. Nevertheless, the highest increased in O^2−^ content was seen by fourfold NaCl + AP-5 groups. Moreover, in O^2−^ content, a threefold increase was determined in the NaCl + DNQX group while a 65% increase in the NaCl + cPTIO group as compared to the control group. When compared to salt application alone, it was observed that the O^2−^ content increased by 20% with DNQX application and 40% with AP-5 application but despite O^2−^ content decreasing with cPTIO application, no significant difference was detected, in the presence of salt (Fig. 5).

### SOD activity

In order to determine how the change in the amount of GLR-mediated NO affects the antioxidant mechanism and glutathione pool; SOD, CAT, POX, CWPOX, APX, GR, MDHAR, DHAR activities and GSH/GSSG amount were determined.

**Fig. 6:**
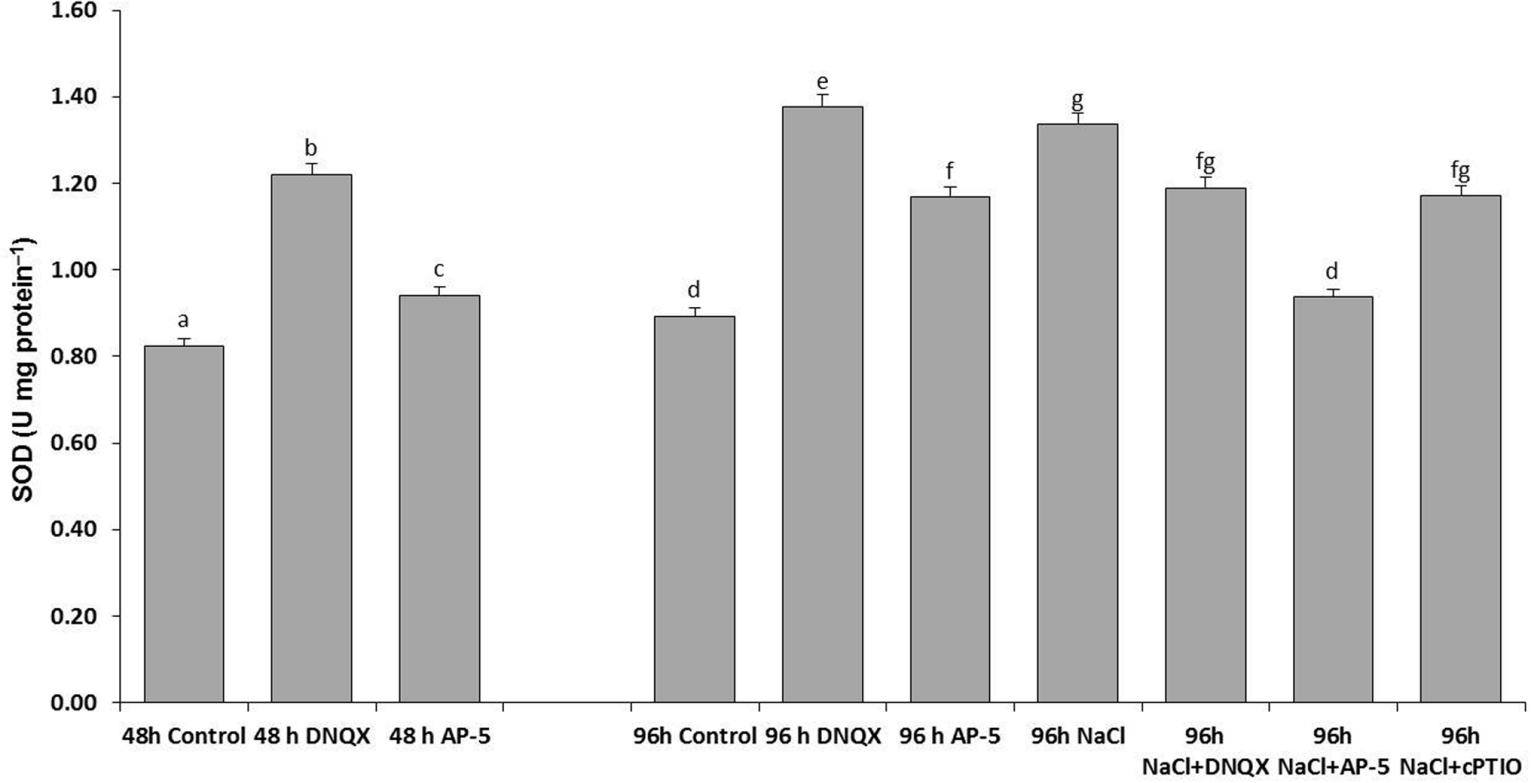
Effect of salt stress (100 mM NaCl), GLR antagonists (0.5 mM DNQX and 0.1 mM AP-5) and NO scavenger (0.5 mM cPTIO) application on SOD activity of Arabidopsis Col-0 ecotype. Data were analyzed with Jamovi 2.3.21 using Tukey’s multiple-range test at a significance level of *p* < 0.05, and different letters **a**–**d** indicate a significant difference between treatments of Data were expressed as the mean ± standard error of three independent biological replicates

At 48 h and 96 h, SOD activity was increased in DNQX and AP-5-treated plants as compared to that of control. Similarly, SOD activity was increased by 49% in solely NaCl-treated plants as compared to that of control. At 96 h, at the solely DNQX-treated plants, observed SOD activity was at its highest. On the other hand, DNQX, AP-5 and cPTIO treatments caused a significant decrease in salt stressed plants. Even, after AP-5 treatment, SOD activity was decreased to almost control levels (Fig. 6).

### CAT activity

**Fig. 7:**
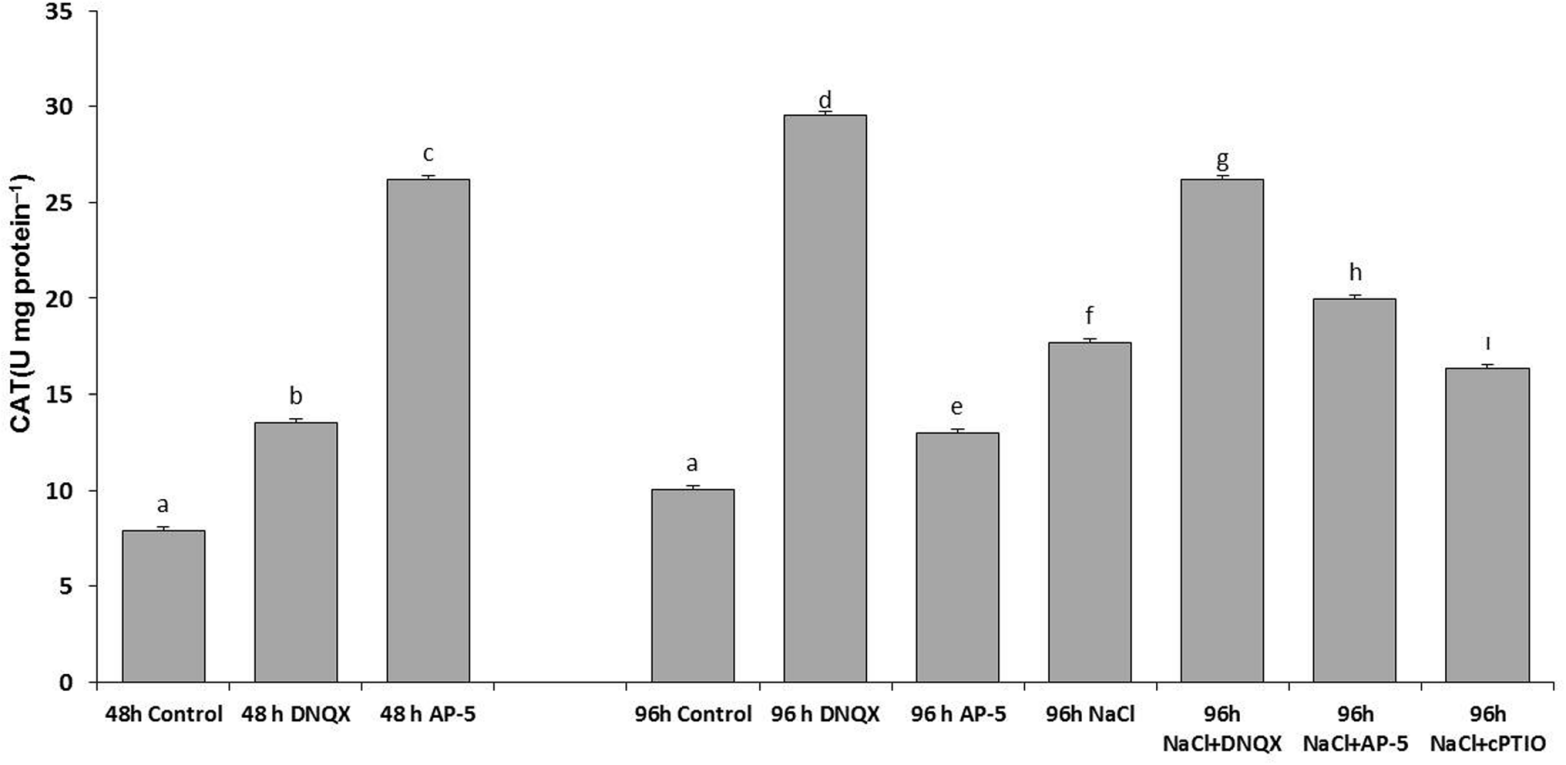
Effect of salt stress (100 mM NaCl), GLR antagonists (0.5 mM DNQX and 0.1 mM AP-5) and NO scavenger (0.5 mM cPTIO) application CAT activity of Arabidopsis Col-0 ecotype. Data were analyzed with Jamovi 2.3.21 using Tukey’s multiple-range test at a significance level of *p* < 0.05, and different letters **a**–**d** indicate a significant difference between treatments of Data were expressed as the mean ± standard error of three independent biological replicates

CAT activity was increased in all groups. The highest increment rate in CAT activity was observed as approximately 3.5-fold and threefold in solely AP-5- treated plants at 48 h and solely DNQX-treated plants at 96 h, respectively as compared to those of control. On the other hand, NaCl treatment increased the CAT activity by only 77%, as compared to control. However, the combination of salinity and DNQX caused a 2.5-fold increase in its activity while the combination of salinity and AP-5 increased the CAT activity by 98%, as compared to control. Moreover, CAT activity was increased by 62% in NaCl + cPTIO-treated plants as compared to that of control (Fig. 7).

### POX activity

**Fig. 8:**
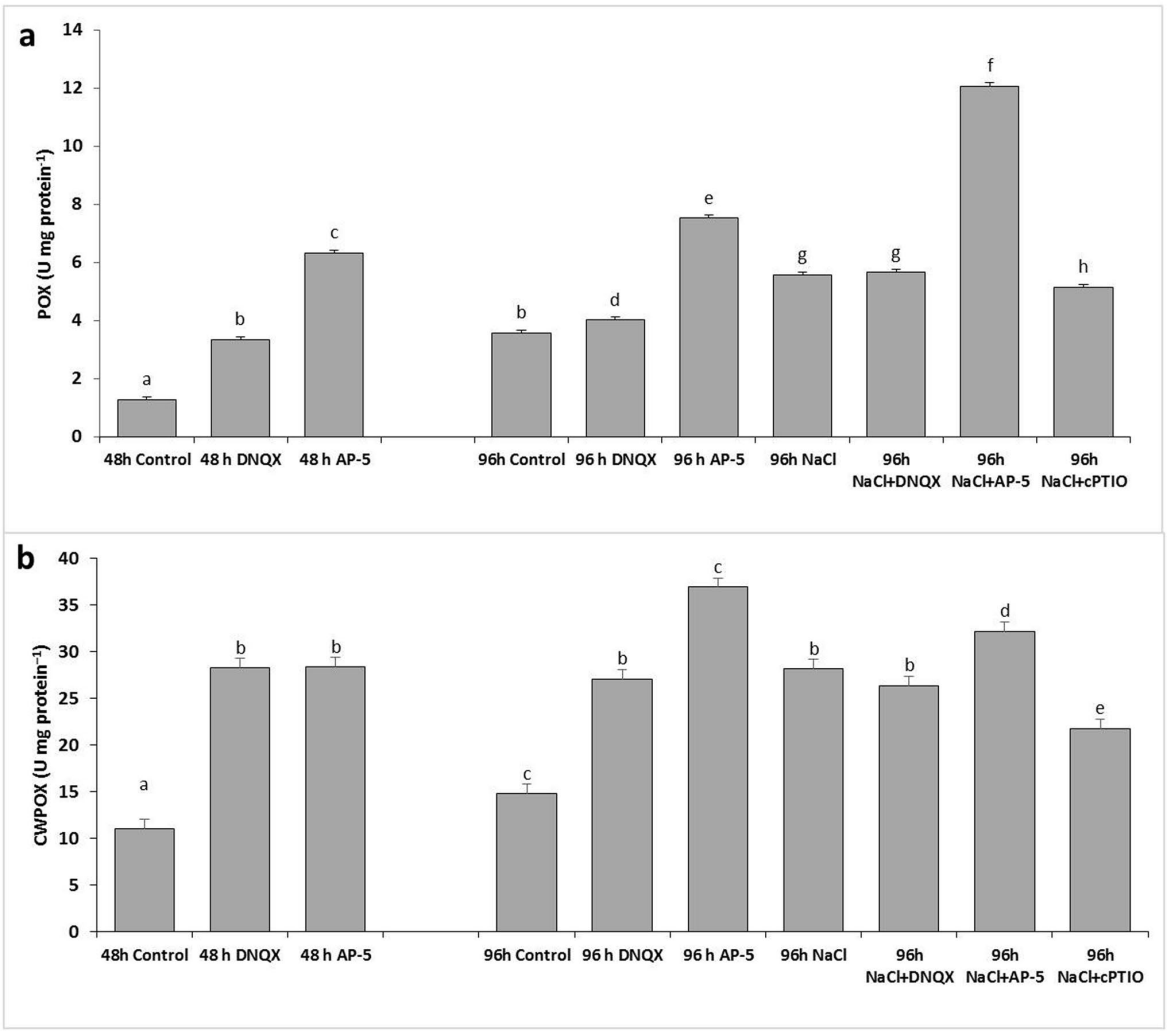
Effect of salt stress (100 mM NaCl), GLR antagonists (0.5 mM DNQX and 0.1 mM AP-5) and NO scavenger (0.5 mM cPTIO) application on **a** peroxidase (POX) and **b** cell wall peroxidase (CWPOX) activity of Arabidopsis Col-0 ecotype. Data were analyzed with Jamovi 2.3.21 using Tukey’s multiple-range test at a significance level of *p* < 0.05, and different letters **a**–**d** indicate a significant difference between treatments of Data were expressed as the mean ± standard error of three independent biological replicates

At 48 h and 96 h, POX activity increased in plants treated with AP-5 and DNQX. Moreover, at 96 h, its activity increased by 56% in solely NaCl-treated plants. Salt stress has escalated POX levels when co-treated with DNQX, AP-5 and cPTIO. The highest increment of POX activity was 2.3 fold when plants were treated with NaCl + AP-5 (Fig. 8a).

### CWPOX activity

At 48 h, DNQX and AP-5 caused an approximately 2.5 fold increase in CWPOX activity. At also 96 h, POX activity was increased by 1.82 fold and 2.5 fold in solely DNQX- and solely AP-5-treated plants as compared to that of control. NaCl treatment increased the CWPOX activity by 90%, as compared to control. However, DNQX, AP-5 and cPTIO treatments under salt stress resulted in higher levels of CWPOX activity. Accordingly, the highest CWPOX activity was detected in NaCl + AP-5 treated plants (Fig. 8b).

### APX activity

**Fig. 9:**
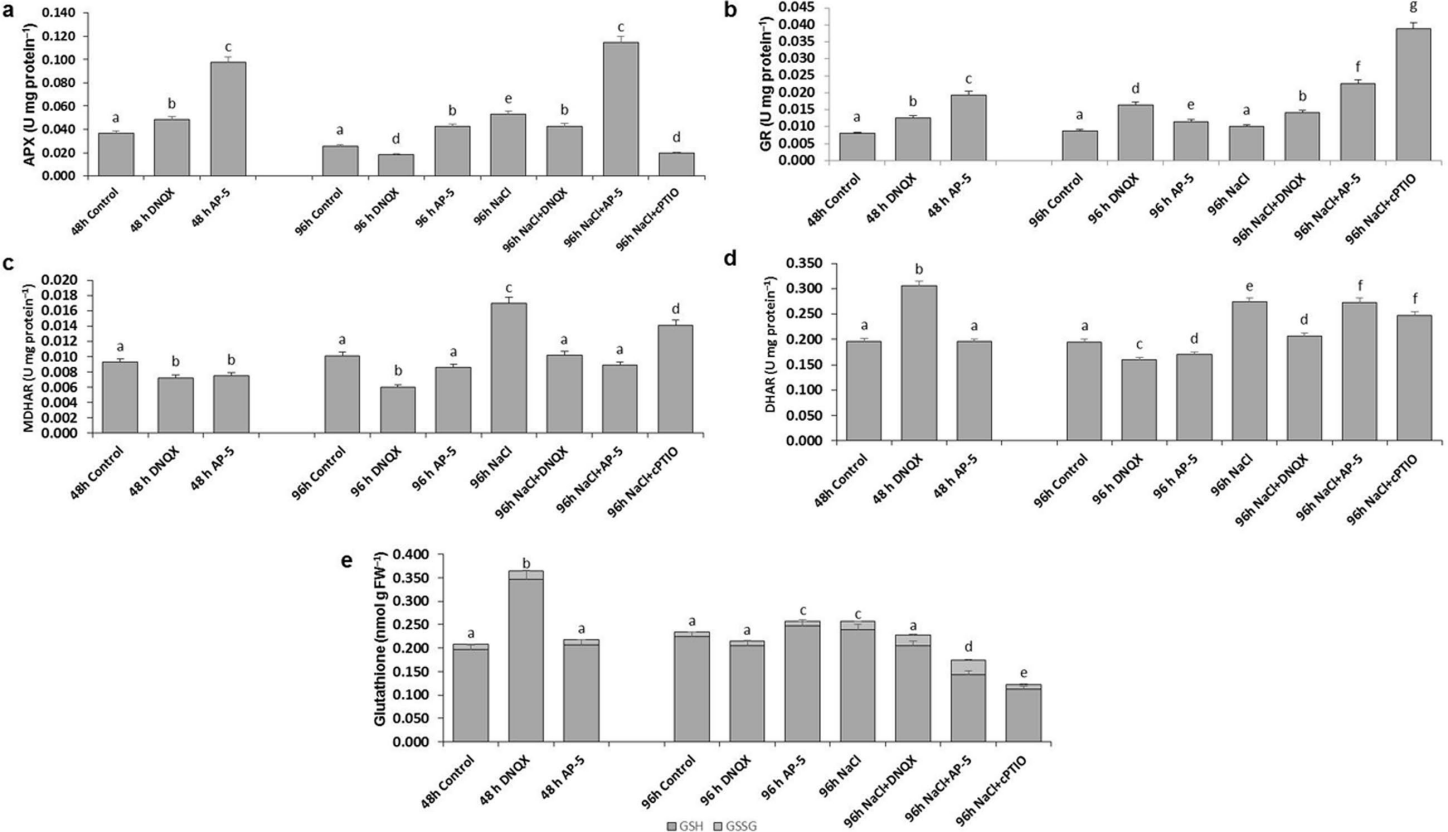
Effect of salt stress (100 mM NaCl), GLR antagonists (0.5 mM DNQX and 0.1 mM AP-5) and NO scavenger (0.5 mM cPTIO) application on **a** ascorbate peroxidase (APOX), **b** glutathione reductase (GR), **c** monodehydroascorbate reductase (MDHAR), **d** dehydroascorbate reductase (DHAR), and **e** glutathione activities of Arabidopsis Col-0 ecotype. Data were analyzed with Jamovi 2.3.21 using Tukey’s multiple-range test at a significance level of *p* < 0.05, and different letters (**a**–**d**) indicate a significant difference between treatments of Data were expressed as the mean ± standard error of three independent biological replicates

At 48 h, DNQX increased the APX activity by 31%, but at 96 h, it caused a significant decrease in APX activity, as compared to control. However, AP-5 increased its activity at both times. APX activity was increased by 100% in the solely NaCl-treated plants at 96 h as compared to that of control. Moreover, combined NaCl and AP-5 caused a higher increase, 338%, in APX activity. However, DNQX increased the APX activity by 65% while cPTIO decreased its activity by 27% (Fig. 9a).

### MDHAR activity

At 48 h, MDHAR activity was decreased by 20% in DNQX and AP-5-treated plants. At 96 h, DNQX continued to decrease the MDHAR activity, but AP-5 did not change its activity. Salt stress resulted in significant increase in the MDHAR activity as compared to control. However, DNQX and AP5 treatments did not change the MDHAR activity in plants under salt stress. Moreover, cPTIO + NaCl increased the MDHAR activity by 38%, as compared to control (Fig. 9c).

### DHAR activity

DHAR activity increased by 55% in the DNQX-treated plants at 48 h as compared to that of control but, it remained at control level in AP-5-treated plants. At 96 h, DNQX and AP-5 caused a significant decrease in DHAR activity of 18% and 12%, respectively, as compared to control. However, its activity was increased by 40% with NaCl application. On the other hand, DNQX (24%) and cPTIO (10%) treatment decreased DHAR activity in the salt-stressed plants, as compared to that of solely NaCl-treated plants, in contrast, AP-5 treatment did not cause any change at DHAR activity (Fig. 9d).

### GR activity

At 48 h, DNQX and AP-5 increased the GR activity. Similarly, at also 96 h, its activity was increased by 77% and 11% in the solely DNQX-treated plants and solely AP-5-treated plants. Nonetheless, salt stress did not change the GR activity, but its activity was increased by 56%, 156% and 333% when DNQX, AP-5 and cPTIO were treated to plants under salt stress (Fig. 9b).

### GSH/GSSG contents

Total glutathione content increased by 75% DNQX-treated plants at 48 h as compared to that of control, this increase was mediated by GSH amount. At 96 h, DNQX did not change total glutathione content, but AP-5 AP-5 increased its activity (10%). In addition, its activity was increased by 10% with NaCl application. On the other hand, DNQX and AP-5 caused a significant decrease in Total glutathione content by 11% and 32%, respectively, in the salt-stressed plants, as compared to that of solely NaCl-treated plants. Moreover, total glutathione content was increased by 52% in NaCl + cPTIO-treated plants as compared to that of solely NaCl-treated plants. (Fig. 9e).

### Lipid peroxidation

**Fig. 10:**
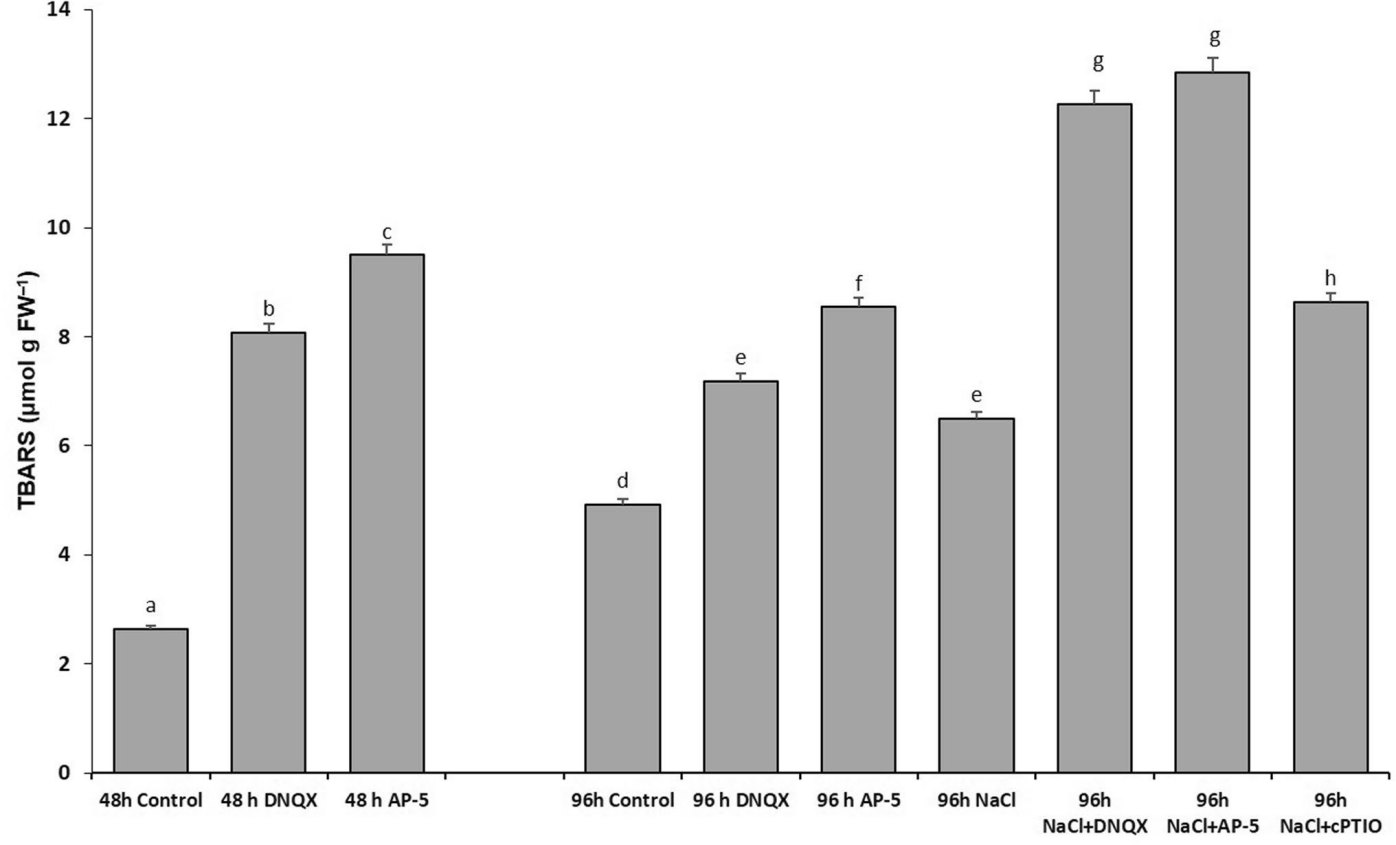
Effect of salt stress (100 mM NaCl), GLR antagonists (0.5 mM DNQX and 0.1 mM AP-5) and NO scavenger (0.5 mM cPTIO) application on the thiobarbituric-acid-reactive substance (TBARS) of Arabidopsis Col-0 ecotype. Data were analyzed with Jamovi 2.3.21 using Tukey’s multiple-range test at a significance level of *p* < 0.05, and different letters (**a**–**d**) indicate a significant difference between treatments of Data were expressed as the mean ± standard error of three independent biological replicates

One of the most important stress markers in plants is lipid peroxidation. For this reason, the change in the TBARS content was determined. At 48 h, the TBARS content was increased by fourfold and 4, fivefold in DNQX and AP-5 treated-plants, respectively, as compared to that of control. At 96 h, the TBARS content was increased by 30% in salt-stressed plants; however, salt-stressed plants in combination with DNQX and AP-5 have exhibited higher increase in TBARS content as compared to that of control. The content of TBARS with antagonist treatments (DNQX and AP-5) increased by approximately 88% and 90%, respectively, compared to salt application alone. This was seen with a 32% increase in cPTIO application in the presence of salt (Fig. 10).

### GSNOR activity

**Fig. 11:**
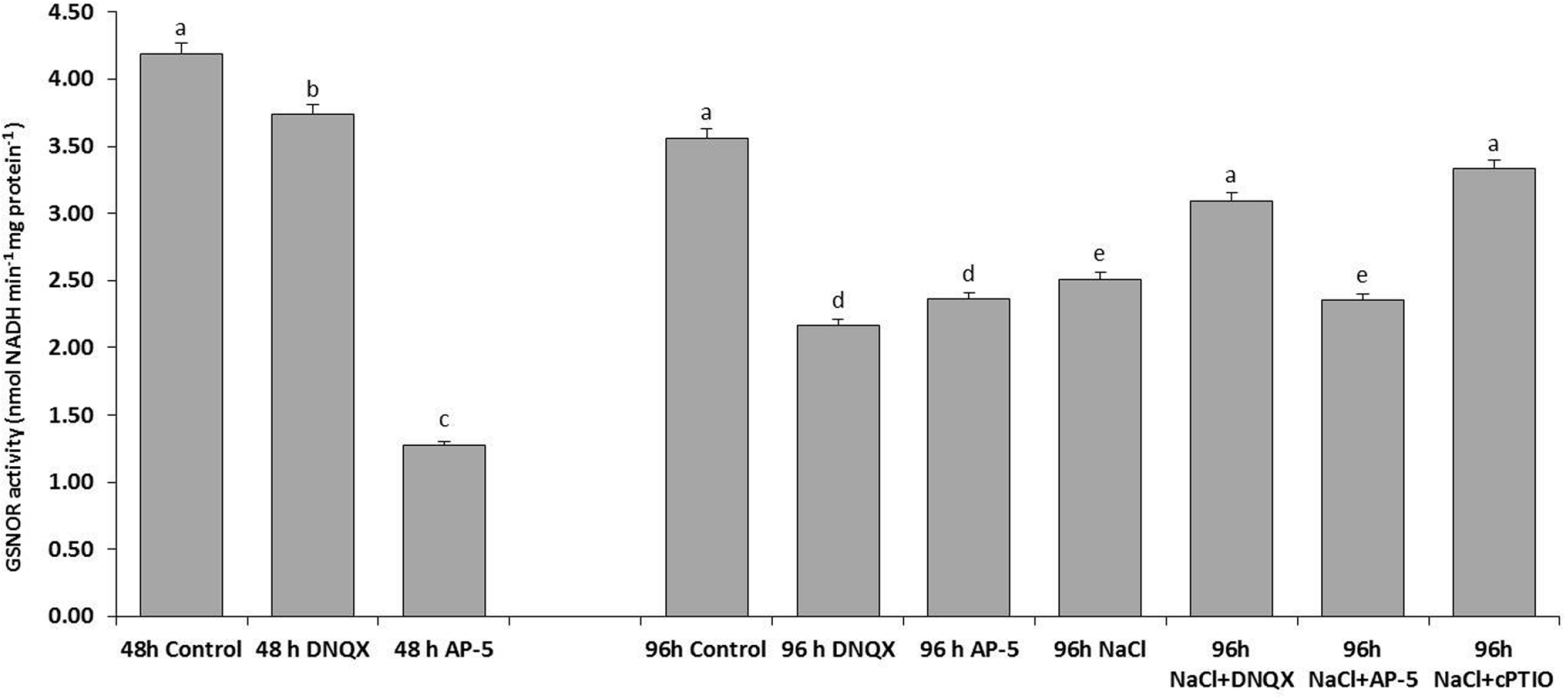
Effect of salt stress (100 mM NaCl), GLR antagonists (0.5 mM DNQX and 0.1 mM AP-5) and NO scavenger (0.5 mM cPTIO) application on GSNOR activity of Arabidopsis Col-0 ecotype. Data were analyzed with Jamovi 2.3.21 using Tukey’s multiple-range test at a significance level of *p* < 0.05, and different letters (**a**–**d**) indicate a significant difference between treatments of Data were expressed as the mean ± standard error of three independent biological replicates

GSNOR, found in plant cells under salt stress, GSNOR activity was determined because it has an important role in limiting or alleviating NO levels in combating stress. At 48 h, GSNOR activity was decreased by 10% and 69% in the DNQX and AP-5-treated plants, respectively as compared to that of control. Similarly, at also 96 h, GSNOR activity decreased by approximately 35% in the solely DNQX-and solely AP-5-treated plants. Only salt stress decreased GSNOR activity by 29% compared to control. The level of GSNOR activity in solely NaCl-treated plants was at the same level with NaCl + AP-5-treated plants. However, DNQX and cPTIO treatments caused a significant increase (23% and 32%, respectively) in plants under salt stress (Fig. 11).

## Discussion

The perception by plants of abiotic and biotic signals induces intercellular signalling networks, involving the activation of ion channels. Among these signaling players, plant glutamate receptor-like channels (GLRs) have emerged as intriguing candidates for sensing environmental clues. Although not much is known about the physicochemical, functional and structural properties of AtGLRs, they were examined in silico in a recent study (Roy and Mukherjee 2017). Moreover, exon–intron structures revealed that the majority of these genes have a common origin, the presence of many phosphorylation and myristoylation sites revealed that AtGLRs participate in various signaling processes, and gene ontology analysis also revealed the participation of AtGLRs in various biological processes, including different stress responses (Roy and Mukherjee 2017).

Studies have highlighted the significant resemblance between Arabidopsis GLRs and animal ionotropic glutamate receptors (iGluRs), particularly in inducing glutamate-mediated calcium signaling (Dennison and Spalding 2000). Recent investigations have shown the involvement of GLRs in cytosolic Ca^2+^ ([Ca^2+^]_cyt_) activity modulation in response to salt stress in Arabidopsis seedlings. Treatment with GLR competitive antagonists inhibited the elevation of [Ca^2+^]_cyt_ activity induced by salt stress, while mutants lacking specific GLRs showed impaired [Ca2 +]cyt elevation in response to NaCl (Cheng et al. 2018). The silico studies highlight AT1G05200’s (AtGLR 3.4) crucial role in rapid environmental stress transmission via Ca^2+^ and its presence in the plasma membrane and plastids. Notably, it demonstrates higher expression in dark conditions and reduced expression under different biotic stresses, suggesting its role in receiving environmental signals through leaves and mitigating stress by downregulating its expression (Roy and Mukherjee 2017). Furthermore, the AtGLR3.4–1 and AtGLR3.4–2 mutant plants were more sensitive to NaCl, compared with the wild-type plants, due to lack of NaCl-induced activation of [Ca^2+^]_cyt_ leading to reduced expression of salt overlay sensitive (SOS) pathway genes and increased accumulation of Na + ions (Cheng et al. 2018). In addition to these, in wild type seedlings, NaCl-induced increase in [Ca^2+^]_cyt_ was prevented by GLR antagonist, 6,7-dinitroquinoxaline-2,3-dione (DNQX). GLRs potentially contribute to Ca^2+^ signal generation, thereby regulating the SOS pathway, a core signaling module responding to salt stress, which involves Ca^2+^ sensor proteins (SOS3 and SOS-like calcium-binding proteins), protein kinase SOS2, and antiporter SOS1 (NHX/H +) (Manishankar et al. 2018). Furthermore, the activation of GLRs in tobacco cells by the fungal elicitor cryptogein triggered calcium influx and a rise in [Ca^2+^]_cyt_, dependent on glutamate release in the apoplast through exocytosis following an increase in [Ca^2+^]_cyt_ (Vatsa et al. 2011). It is interesting that putative GLRs were also found to be taking place in cryptogein-induced nitric oxide (NO) production (Vatsa et al. 2011). While evidence suggests that NO mediates Ca^2+^ signaling, it is also recognized that NO production is contributed by Ca^2+^ fluxes. Notably, extracellular Ca^2+^ uptake has been shown to improve NO accumulation in guard cells of Arabidopsis epidermis, leading to calcium sensing receptor-regulated stomatal closure (Wang et al. 2012). Recently, Philippe et al. (2019) found the increase in NO levels is dependent on GLR activation in *Medicago truncatula* leaves under short-term water deficit stress. These collective findings have also convincingly shown the significance of glutamate receptor-like channels in plant signaling pathways, particularly in relation to calcium and nitric oxide signaling. Our study presents a novel hypothesis suggesting that modulating the antioxidant defence by salt stress-induced nitric oxide (NO) production in Arabidopsis Col-0 seedlings is mediated by putative glutamate receptors (GLRs). To test this hypothesis, we conducted subsequent experiments utilizing various antagonists and scavengers, including the GLR competitive antagonists (D-2-amino-5-phosphono pentanoic acid) AP-5 and DNQX, which are the most commonly used agents as NMDA and AMPA glutamate receptor antagonists, respectively, as well as the NO scavenger cPTIO. Inhibitory effect of DNQX on all AtGLRs is assumed to rely on binding within the ligand binding sites, whereas AP-5 is known to compete with the natural ligand, possibly at the L-glutamate binding site (Dubos et al. 2003). Our results demonstrated that the elevated NO content induced by salt stress was effectively suppressed in the leaves of AP-5 and DNQX-treated Arabidopsis plants under salt stress (Fig. 2). However, the highest suppression occurred with DNQX. Similarly, a decrease in NO content was noted also with Arabidopsis leaves treated with cPTIO. These findings provide compelling data for a connection between putative AtGLR and NO signaling pathways under salt stress conditions.

Salt stress triggers osmotic stress, ion toxicity and oxidative stress that limit plant survival, biomass production and development (Lelandais-Brière et al. 2007). The inhibition of plant growth under salt stress has been documented in numerous studies (Rahman et al. 2017; Shahid et al. 2020; Zhao et al. 2021). Consistent with these findings, our study revealed a significant inhibition in the shoot fresh weight of Arabidopsis Col-0 under salt stress. Furthermore, we observed that the additions of AP-5 and DNQX further enhanced the inhibition of Arabidopsis shoot growth in response to NaCl-induced salt stress. These observations underscore the critical role of AtGLRs and their involvement in NO production which are essential for shoot development under salinity. One of the first responses of all plants to drought or salt-induced osmotic stress is a reduction in relative water content triggered by reduction of leaf turgor/water potential (Guerfel et al. 2008) (Table 1). Similarly, salinity decreased RWC in our study. In addition, while the amount of RWC did not change in the groups exposed to AP-5, a decrease was observed in DNQX. However, the presence of DNQX and AP-5 under salt stress led to a decrease, just as in cPTIO. This showed that the suppression of GLR-mediated NO by antagonists made it difficult for the plant to maintain its water status (Table 1).

Previous studies have established that salt stress leads to the reactive oxygen species (ROS) production through the activation of the plant plasma membrane NADPH oxidase (NOX) (Kurusu et al. 2015). However, it has been demonstrated that NO suppresses NADPH oxidase-dependent ROS production by means of S-nitrosylation in both human endothelial cells (Selemidis et al. 2008) and plants (Yun et al. 2011). In our study, we observed that GLR antagonists effectively induced the NOX activity in plants subjected to salt stress as well as cPTIO-treated plants. This inhibitory effect of putative AtGLRs on salt stress-induced NOX activity can be attributed to the induction of NO levels which is dependent on GLR activation in Arabidopsis leaves under salt stress.

In addition to these, our investigation revealed that treatments with AP-5 and DNQX (GLR compatative antagonists) and cPTIO significantly increased the contents of ROS, such as H_2_O_2_ and O^2.−^, in Arabidopsis leaves subjected to salt stress, for example, they increased the H_2_O_2_ content approximately by 25% at 96 h, compared with solely salt-treated plants. Furthermore, O^2−^ content was increased by fourfold, threefold, and 65% in the AP-5, DNQX and cPTIO-treated plants under salt stress, respectively. Accordingly, we demonstrated for the first time that putative AtGLRs, through the production of NO leading to the inactivation of NOX, play a crucial role in regulating the ROS content under salt stress conditions.

Studies have yet to determine whether increase in NO levels, which is dependent on GLR activation, have alleviated NaCl-induced oxidative stress damage in Arabidopsis seedlings. In this study, the decreased SOD activity in salt-stressed plants with AP-5, DNQX and cPTIO pretreatment could likely decrease the plant’s response capacity to maintain O^2−^ homeostasis. On the other hand, we found that AP-5 and DNQX significantly increased the activities of H_2_O_2_ -scavenging enzymes, such as peroxidase (POX), cell wall-bound peroxidase (CWPOX) and catalase (CAT) in Arabidopsis leaves under salt stress compared with that in the salt-treated plants alone; however, when Arabidopsis seedlings were treated with a NO scavenger (cPTIO) in the presence of NaCl, the induced activities of CAT, POX and CWPOX were significantly inhibited. This inhibition aligns with previous reports by Fan et al. (2007), Guo et al. (2009), López-Carrión et al. (2008), Sheokand et al. (2008), Shi et al. (2007) and Yu-qing et al. (2007). We speculated that the activities of H_2_O_2_ -scavenging enzymes induced by salt treatment were not dependent on AtGLR-mediated NO production.

APX, GR, DHAR and MDHAR are enzymes involved in the ascorbate–glutathione cycle, which plays a crucial role in scavenging H_2_O_2_. The activities of these enzymes showed significant decrease after treatment with cPTIO under salt stress. The results illustrated that NO was responsible for the activities of ASC-GSH cycle enzymes. NO has been indicated to induce antioxidant enzyme activities via activating mitogen-activated protein kinase cascade in maize (Zhang et al. 2007). Similarly, under salt stress conditions, the activities of APX, GR, DHAR and MDHAR were markedly diminished in plants pretreated with AP-5 and DNQX. It has been shown that DNQX and AP-5 have a similar mode of action on the ASC-GSH cycle by changing the amount of GLR-mediated NO. As a consequence, there was an increase in GSSG content and a decrease in the GSH/GSSG ratio (Fig. 9), which hindered the effective removal of ROS and resulted in excessive ROS accumulation (Figs. 4, 5). This ultimately led to the impairment of the cell membrane (Fig. 10). In addition, similar activities were observed compared with those treated by cPTIO. The data shows that putative AtGLRs, through NO production, are required for induction of the ascorbate–glutathione cycle indicating the important role of glutamate receptors in the salt stress tolerance. AP-5 and DNQX treatments decreased the salt-induced oxidative stress tolerance of Arabidopsis plants mainly through the SOD and ASC-GSH cycle indicating that the increase in NO levels, which is dependent on GLR activation, stimulated the stress response system of Arabidopsis plants. On the other hand, when the effects of both antagonists on the activity of antioxidant enzymes in the leaves of the plant under salt stress were examined, it was determined that DNQX significantly suppressed the activities of SOD, DHAR, and APX to a greater extent than AP-5 due to the mode of action of DNQX in the cell.

In the presence of O^2−^, NO reacts with reduced glutathione (GSH) to generate S-nitrosoglutathione (GSNO), which acts as a NO donor (Fancy et al. 2017). GSNO is then subjected to NADH-dependent denitrosylation by the enzyme GSNO reductase (GSNOR), resulting in the formation of oxidized glutathione (GSSG) and NH3. Therefore, GSNOR in plant cells under salt stress conditions has a crucial role in limiting or alleviating NO levels to combat stress (Hasanuzzaman et al. 2013). Previous studies showed that under salt stress, plants enhance the synthesis of nitric oxide (NO) primarily by upregulating a NOS-like enzyme activity or nitrate reductase (NR) while concurrently suppressing the activity of GSNOR (Shang et al. 2022). In the present study, antagonists and scavengers caused a significant increase in GSNOR activity in the salt-stressed plant’s leaves compared with those of solely salt-treated plants thereby resulting in a high GSSG content and low GSH/GSSG ratio (Fig. 9). These results suggest that putative AtGLRs could be key players in maintaining the cellular redox homeostasis under salt stress by regulating the GSH/GSSG ratio.

## Conclusions

In conclusion, our study represents pioneering research in Arabidopsis, revealing the significant role of GLR-mediated nitric oxide (NO) production in safeguarding leaf tissues against salt stress. Our findings demonstrate the crucial involvement of GLR-mediated NO in preserving leaf functionality under salt stress conditions by reducing the accumulation of reactive oxygen species (ROS) and increasing the activity of SOD and the ASC-GSH cycle. These mechanisms effectively contribute to maintaining membrane integrity by reducing lipid peroxidation. However, further laboratory experiments are needed based on the comparative analysis and expression analysis results elucidating the chromosomal positions, gene structures, and phylogenetic relationships of AtGLR genes to comprehend the intricate mechanisms of GLR-mediated signaling. Advanced research in this field will expand our knowledge and strengthen potential applications in enhancing stress tolerance and agricultural productivity.

## Data Availability

The raw data required to reproduce the above findings cannot be shared at this time as the data also forms part of an ongoing study.
